# Dynamics of HIV-Containing Compartments in Macrophages Reveal Sequestration of Virions and Transient Surface Connections

**DOI:** 10.1371/journal.pone.0069450

**Published:** 2013-07-29

**Authors:** Raphaël Gaudin, Stefano Berre, Bruna Cunha de Alencar, Jérémie Decalf, Michael Schindler, François-Xavier Gobert, Mabel Jouve, Philippe Benaroch

**Affiliations:** 1 Institut Curie, Centre de Recherche, Paris, France; 2 INSERM U932, Immunity and Cancer, Paris, France; 3 CNRS UMR 3215/INSERM U934, Paris, France; 4 Institute of Virology, Helmholtz Zentrum Munich, Munchen, Germany; New York University, United States of America

## Abstract

During HIV pathogenesis, infected macrophages behave as “viral reservoirs” that accumulate and retain virions within dedicated internal Virus-Containing Compartments (VCCs). The nature of VCCs remains ill characterized and controversial. Using wild-type HIV-1 and a replication-competent HIV-1 carrying GFP internal to the Gag precursor, we analyzed the biogenesis and evolution of VCCs in primary human macrophages. VCCs appear roughly 14 hours after viral protein synthesis is detected, initially contain few motile viral particles, and then mature to fill up with virions that become packed and immobile. The amount of intracellular Gag, the proportion of dense VCCs, and the density of viral particles in their lumen increased with time post-infection. In contrast, the secretion of virions, their infectivity and their transmission to T cells decreased overtime, suggesting that HIV-infected macrophages tend to pack and retain newly formed virions into dense compartments. A minor proportion of VCCs remains connected to the plasma membrane overtime. Surprisingly, live cell imaging combined with correlative light and electron microscopy revealed that such connections can be transient, highlighting their dynamic nature. Together, our results shed light on the late phases of the HIV-1 cycle and reveal some of its macrophage specific features.

## Introduction

Macrophages are versatile cells present in most tissues and endowed with a variety of functions, including innate and adaptive immunity. Macrophages are targets of HIV that play a key role in several aspects of AIDS pathogenesis. Macrophages also serve as long-lived reservoirs of virus that appear resistant to drug treatments and to attacks from the immune system [1], [2], [3], [4]. Despite these important physiopathological features, little is known about the HIV replication cycle in macrophages, especially regarding viral assembly.

Infected macrophages accumulate large internal vacuoles containing virus. These Virus-Containing Compartments or VCCs are not found in infected T cells. Several studies have established that newly formed viral particles bud and pinch off at the limiting membrane of the VCCs [5], [6], [7], thus accumulating virions in the lumen of the compartment. Although their exact nature remains debated [8], [9], VCCs do not seem to be part of the endocytic pathway as they are not acidified and remain inaccessible to exogenously added BSA-gold [10], [11]. VCCs contain a subset of markers (such as CD9, CD81, CD44 and CD18) that are present in endosomes but can also traffic to the plasma membrane [6], [12]. However, VCCs appear poor or free of some other endosomal markers such as EEA1 and LAMP1 [8]. At the ultrastructural level, VCCs frequently exhibit a thick molecular coat at their limiting membrane that may contain ß2 integrins, cytoskeletal linker proteins [12] and members of the ESCRT machinery [9]. We recently showed that VCCs are spatially organized along the microtubule network on which the molecular motor KIF3A participate to their motion towards the cell periphery. The kinesin KIF3A is thus critical in HIV-1-infected primary macrophages for the production of viral particles [13]. Finally, some VCCs were found to be connected to the plasma membrane through microchannels too narrow to allow the passage of virions [14]. It was then proposed that VCCs originate from the plasma membrane and may actually correspond to membrane domains deeply sequestered within the cell [11], [14]. Although attractive, the hypothesis of the plasma membrane origin of VCCs still lacks direct demonstration. Consequently, the nature of VCCs is still controversial and their function remains unknown.

The aim of the present study was to approach the function of the VCCs by studying their dynamics in primary macrophages upon HIV-1 infection. Taken together, our data suggest a mechanism of progressive sequestration of viral particles in intracellular VCCs, thus reducing the amount of virus secreted with time. They shed light on the complexity of VCC dynamics and maturation in primary macrophages. Finally, our results are compatible with the plasma membrane origin of VCCs.

## Results

### Analysis of the Virus-Containing Compartment Dynamics Reveals their Heterogeneity

Since HIV-1 Gag is a structural polyprotein critical for viral assembly, we investigated the kinetics of its expression and localization in HIV-infected macrophages. For this purpose, we used HIV Gag-iGFP, a HIV-1 coding for a fusion protein with a GFP inserted between the Matrix and the Capsid domains of Gag, flanked by protease sites [15] and carrying a macrophage-tropic Env. HIV Gag-iGFP virus was efficiently produced in 293T cells without the help of wild type Gag and was compared to its untagged HIV WT virus version on primary macrophages. Both viruses were comparable in terms of kinetics of p24 production in the supernatant (Fig. S1A) and intracellular localization of Gag into Env+ CD81+ Lamp1- intracellular compartments (Fig. S1B and C). Although lesser than its WT counterpart, HIV Gag-iGFP was infectious (Fig. S1D), demonstrating that the virus cycle was properly completed and suitable for use on primary macrophages.

To test whether GFP fluorescence visualized in the cytosol of HIV Gag-iGFP-infected macrophages reflected true Gag-iGFP or free GFP following a putative early cleavage, we took advantage of the capacity of free GFP to accumulate in the nucleus. Infection of primary macrophages for 7 days with a GFP encoding lentivector led to GFP concentration in the nucleus as expected (Fig. S2). In contrast, no GFP was detected in the nuclei of macrophages infected with HIV Gag-iGFP for 7 days, whereas large Gag-iGFP+ compartments accumulated in the cytosol (Fig. S2). These data suggested that GFP is freed from Gag-iGFP only within the viral particles where it stays trapped. At the ultrastructural level, HIV Gag-iGFP-infected macrophages exhibited typical VCCs with mature and immature viral particles in their lumen (Fig. S1E). The presence of mature particles exhibiting typical conical capsid supported the idea that the Gag-iGFP precursor was efficiently cleaved by the viral protease as shown by immunoblot analysis of infected macrophage lysates (Fig. S1F).

**Figure 1:**
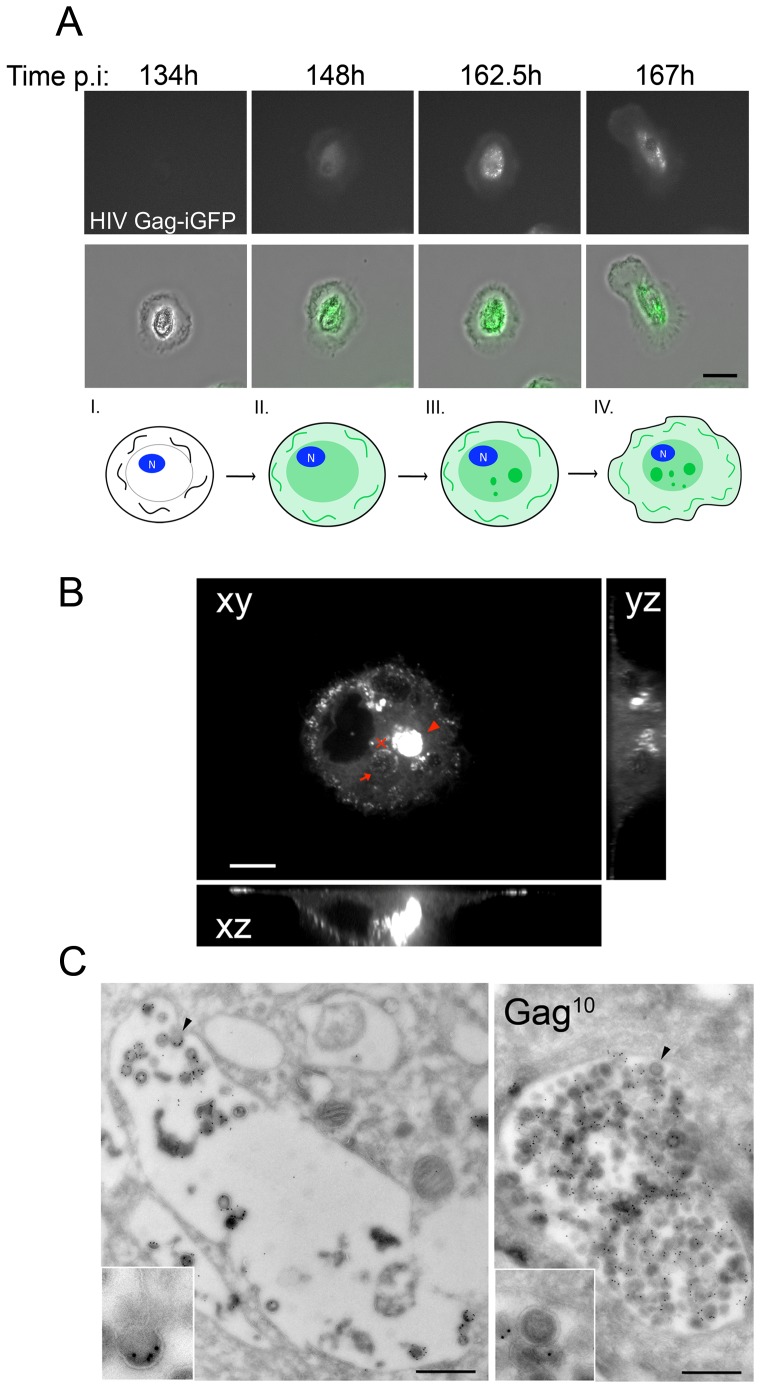
Characterization of the Virus-Containing Compartments. (*A*) Dynamic imaging of the HIV cycle in macrophages. Macrophages infected with HIV Gag-iGFP virus were imaged from 1 to 8 dpi. To minimize photo-cytotoxicity, images were acquired every 15 min with an epifluorescent Biostation microscope (see Supplemental Video S1). Here are presented 4 snapshots from the movie at the indicated times post infection. Epifluorescent and corresponding transmission images are presented. Bar 20 µm. Bottom diagram. Schematic representation of Gag expression in infected macrophages at four morphologically distinct stages corresponding to the images presented above. (I) Gag is not yet expressed (II) Gag is detected in the cytosol in a diffuse pattern (III) Gag concentrates in internal compartments that accumulate and are in motion, and (IV) macrophages become more motile. (*B*) Heterogeneity of the VCCs in primary macrophages. Spinning disk confocal micrograph of the central region of a macrophage infected for 5 days with HIV Gag-iGFP ΔEnv. Orthogonal plans (xz and yz) from the red cross are also presented. The arrow points to a compartment that, by time-lapse analysis (see corresponding Supplemental Video S2), contained few Gag+ structures moving fast in the lumen, while the arrowhead indicates a compartment that appeared full and still. Bar 5 µm. (*C*) VCCs exhibit heterogeneous density of virions. Ultrathin cryosections of macrophages infected with HIV-1 NLAD8 for 7 days were prepared and labeled for p17 with protein A coupled to gold 10 nm. Two examples of VCCs are shown, one is packed with virions (right), the other (left) only contains a few virions. Bar 500 nm.

We then established the spatiotemporal distribution of Gag in HIV Gag-iGFP-infected macrophages by time-lapse microscopy for up to 8 days (Fig. 1A and corresponding supplemental Video S1). Gag-iGFP started to be detected as a diffuse staining in the cytosol of macrophages within 1 to 8 dpi. Of note, since we used a replication-competent virus, the precise initial time of infection cannot be determined. To circumvent that problem, we also used a single-cycle virus (HIV Gag-iGFPΔEnv), and observed that cytosolic Gag was visible between 1 and 3 dpi (Fig. S3), a delay probably required given the slow speed at which the first phases of the viral cycle take place in primary macrophages [16]. In both cases, i.e. using Env-deficient or -sufficient viruses, the first Gag+ compartments were visible approximately 0.5 to 1 day after the first detection of cytosolic Gag (14 h30±4 h30; n = 14 movies), indicating that the formation of Gag+ compartments was Env-independent. One day later the cells became more motile exhibiting increased plasma membrane movements, in agreement with previous observations [17]. Thus, following infection of primary macrophages by HIV-1 *in vitro,* four morphologically distinct stages can be sequentially observed (see Fig. 1A, bottom diagram, see legend).

### Heterogeneity of Viral Content in Virus-Containing Compartments

Next, we sought to analyze HIV Gag-iGFP dynamics at a better spatial and temporal resolution using time-lapse analysis by spinning disk confocal microscopy of HIV Gag-iGFP ΔEnv infected macrophages. We observed 2 main types of Gag+ compartments in the central area of infected macrophages (Fig. 1B). Some compartments appeared relatively sparse (see arrow) but contained Gag+ structures moving at high velocity (supplemental Video S2), suggesting that virions or clusters of virions (below the resolution limit) were in motion in these compartments. In contrast, other Gag+ compartments appeared very dense as if they were entirely packed with viral particles (see arrowhead). In this case, no clear movement was detected. These observations were supported by electron microscopy (EM) analysis of macrophages infected with HIV-1 WT showing relatively sparse or dense VCCs exhibiting, in both cases, budding profiles at their limiting membrane (Fig. 1C), indicating that virus assembly can take place within the VCC.

**Figure 2:**
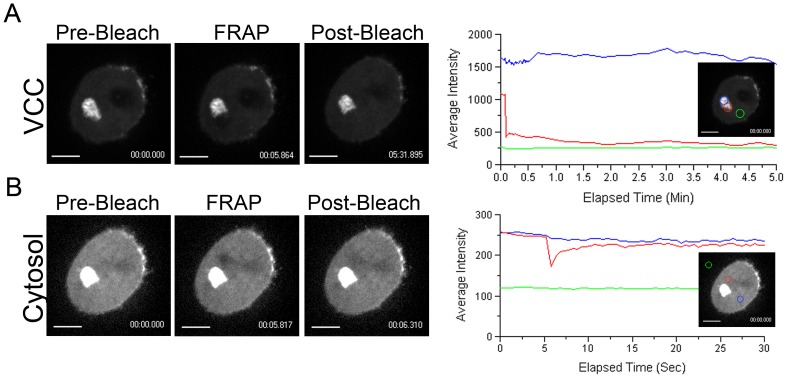
Heterogeneity of the Virus-Containing Compartments. (A–B). Analysis of the VCC by photobleaching experiments. FRAP of Gag-iGFP in the VCC or cytosol of infected macrophages. Live macrophages infected for 4 days with HIV Gag-iGFP ΔEnv were imaged by spinning disk confocal microscopy every sec for 5 sec, then bleached at maximum laser power for 20 msec. Right after photobleaching, images were acquired every 500 ms for 30 sec (panel B, Supplemental Video S4) or 500 ms for 30 sec and then every 10 sec for 5 min (panel A, Supplemental Video S3). On the left panels, images from the Movies represent Gag-iGFP, before, right after, and 5 min (A) or 500 ms (B) after photobleaching. On the right panels, average intensity of the regions of FRAP (red circles and lines) and controls (blue and green circles and lines), are represented as a function of time. Gag-iGFP in the cytosol quickly recovers after photobleaching, which is not the case in VCCs. Bar 5 µm. Data are representative of two independent experiments performed with two donors.

The presence of dense GFP+ compartments suggested that such VCCs were tightly packed with viruses. However, this could also reflect a lack of sufficient spatial resolution rather than stillness of the Gag-iGFP. To discriminate between these two possibilities, we performed Fluorescence Recovery after Photobleaching (FRAP) analysis on such compartments. Strikingly, when Gag-iGFP dense compartments were subjected to photobleaching, no recovery of fluorescence was observed over time (Fig. 2A and corresponding supplemental Video S3), suggesting that Gag-iGFP and thus viral particles are unable to move within such compartments. As a control, we photobleached areas of the cytosol where rapid recovery was observed (half-life approx. 1 sec) (Fig. 2B and corresponding supplemental Video S4). We conclude that “dense” VCCs are packed with viral particles such that they remain rather immobile in the lumen of the compartment.

### Evolution of VCCs with Time Post-infection

**Figure 3:**
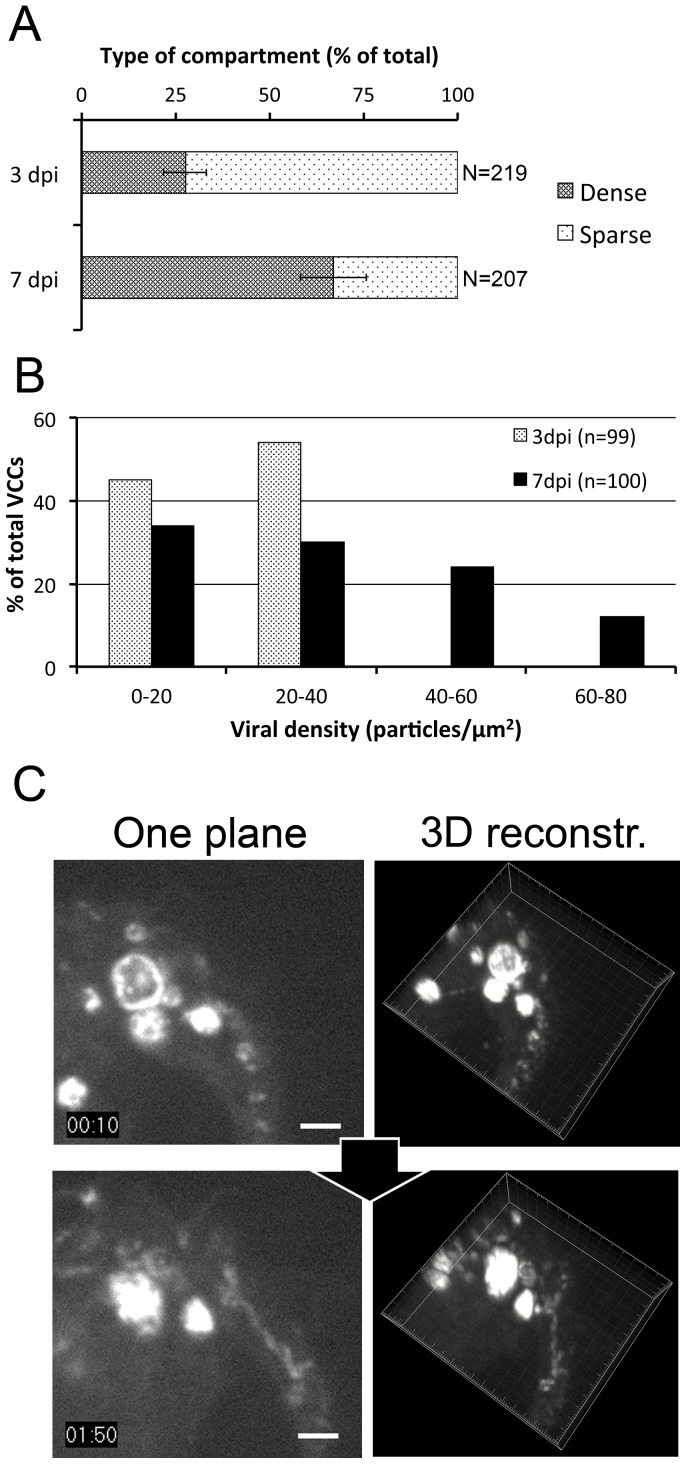
Virus-Containing Compartments fill up with time. (*A*) Quantification of sparse versus dense VCCs at 3 or 7 dpi. Live macrophages infected with HIV Gag-iGFP ΔEnv were visualized by spinning disk confocal microscopy. Taking into account the compartments with a diameter superior to 1 µm, we considered a VCC as sparse when internal movement was detected; otherwise it was counted as a dense VCC. Values presented are means +/− SD of the percentage of sparse versus dense compartments calculated from 4 donors. Statistical significance was calculated for the differences between day 3 and 7 (*p* = 0.0003). (*B*) Quantification of viral density within VCCs at 3 and 7 dpi. Macrophages were infected with HIV NLAD8 and fixed at 3 or 7 dpi. Then, samples were embedded in epon for electron microscopy. For each VCC identified, the size of the VCCs and the number of particles per VCC were quantified. The graph represents the distribution of the viral particle density of the VCCs at both time points. *(C)* Time-lapse imaging of VCCs. Macrophages infected with HIV Gag-iGFP ΔEnv virus were imaged at 4 dpi during 2 h. Three-dimensional images were acquired every 5 min with a spinning disc microscope. Here are presented 2 snapshots (left panels) from the Supplemental Video S5 and their corresponding 3D reconstructions (right panels). A Gag-iGFP+ compartment is slowly filled. Note that two compartments fuse between 1∶30 and 2∶00 h. Bar 2 µm.

To quantify the proportion of the two types of compartments and its evolution, live imaging of HIV Gag-iGFPΔEnv-infected macrophages was performed at 3 or 7 dpi. The ratio of the percentages of sparse/dense VCC was evaluated for 4 donors. This ratio evolved with time: from 72/28% (n = 219) at 3 dpi to 33/67% (n = 207) at 7 dpi (Fig. 3A), suggesting progressive maturation from sparse to dense VCC. Importantly, quantification at the ultrastructural level revealed that the density of viral particles per µm^2^ in VCCs increased with time. Indeed, analysis of VCCs by immuno-EM demonstrated that the density of the compartments drastically shifted from low to high, between 3 and 7 dpi (Fig. 3B).

To study the relationship between the two types of compartments in a dynamic manner, we performed 4D imaging. Macrophages infected with HIV Gag-iGFPΔEnv were imaged using spinning disk confocal microscopy at 3 dpi for 24 hours, 1 stack every 5 min. We observed filling events of Gag+ compartments that became denser with Gag+ material with time (Fig. 3C), further suggesting that sparse compartments mature by accumulating virus in their lumen. We ruled out the possibility that the compartment moved to another plan by 3D reconstruction (Fig. 3C right panels). Of note, we also witnessed events of fusion between compartments (supplemental Video S5), suggesting that homotypic fusion between VCCs can occur. Such fusion events may contribute to the large size of some VCCs observed in HIV-infected macrophages.

We conclude that in HIV-infected macrophages, VCCs begin with a low density of viral particles that appear highly motile. The proportion of dense versus sparse VCCs, as well as their density in viral particles increases with time post infection.

### Sequestration of Viral Particles in Infected Macrophages

**Figure 4:**
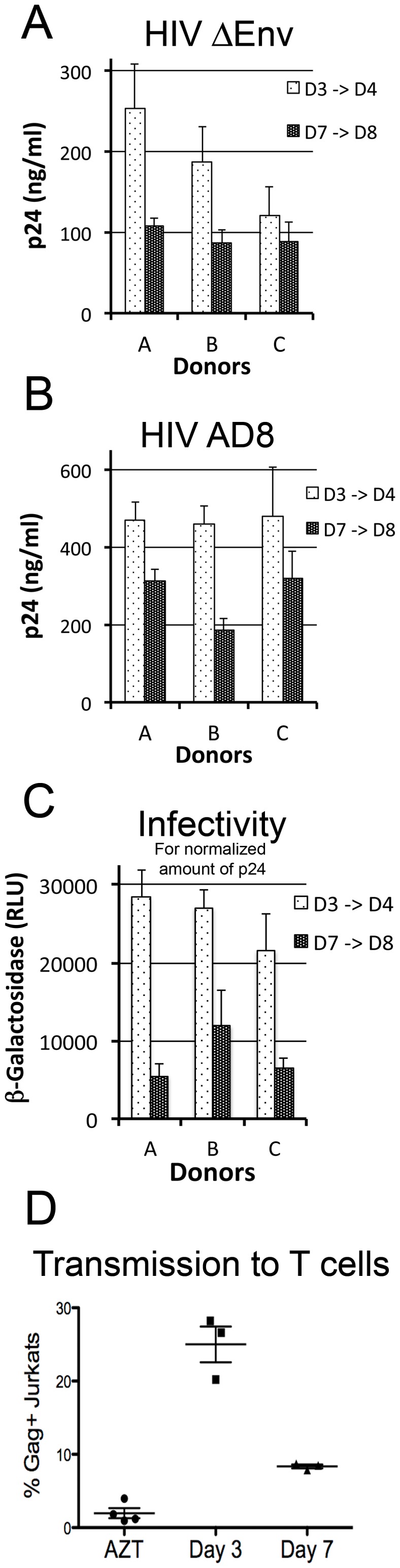
Amount and quality of the viral production as a function of time post-infection. (A–C) Macrophages released less particles at 7 than 3 dpi. Macrophages were infected with either HIV ΔEnv (A) or HIV NLAD8 (B) and washed the day after. Then, p24 released in the supernatant for 24 hours was evaluated between day 3->4 and 7->8 post infection by Elisa. Means of quadruplicate performed for 3 different donors are presented with their SDs. Analysis for 6 donors reveals a decrease between 3 and 7 dpi with statistical significance (*p* = 0.0002). (C) Infectivity of the viral particles released by macrophages at 3 dpi decreases at 7 dpi. Same amount of p24 (0.75 ng) from supernatant collected between day 3->4 and 7->8 post infection were added to TZM-bl reporter cell lines. After 24 hr, ß-Gal activity was measured by luminometry. Viruses produced between day 7 and 8 post infection are less infectious (from 50 to 80% reduction, depending on the donor) than the one produced between day 3 and 4 post infection. (D) Kinetics of macrophage to T cell transmission of HIV. Macrophages infected with HIV-1 for 3 or 7 days were co-cultured with Jurkat T cells for 24 hours and then for another 24 hours in the presence of AZT to allow newly synthesized Gag to accumulate while avoiding transmission between T cells. As control, AZT was added to some wells at the beginning of the co-culture. Cells in suspension were collected, fixed, stained for intracellular p24 and analyzed by flow cytometry. Results are expressed as % of infected (Gag+) Jurkat T cells. One representative experiment out of 3 is presented.

Our results prompted us to further analyze the evolution of the viral production with time post-infection. Surprisingly, monitoring the amount of p24 secreted by macrophages from 3 donors during 24 hr between day 3 and 4, and between day 7 and 8, revealed a strong diminution with time. This was true for a single cycle virus (ΔEnv) (Fig. 4A), as well as a WT virus (AD8) (Fig. 4B). Moreover, while the amounts of p24 released in 24 hr in the supernatant of macrophages infected with NL4-3 ΔEnv decreased from day 3 to 7, they increased in the matched cell lysates, further suggesting that virions were retained into cells (Fig. S4). Furthermore, the infectivity of virions produced by NLAD8-infected macrophages, normalized to a given amount of particles quantified by p24 ELISA, profoundly decreased with time (from 55% to 80% inhibition depending on the donors) (Fig. 4C). Since HIV efficiently propagates through cell-to-cell contacts [18], the capacity of infected macrophages to transmit HIV-1 to T cells was assayed. Macrophages infected with VSV-G pseudotyped HIV-1 NL-4.3 (T-tropic) at 3 or 7 dpi were co-cultured for 24 hr with Jurkat T cells. After an additional 24 hr in the presence of AZT to allow newly synthesized Gag to accumulate, Jurkat cells were recovered and analyzed by flow cytometry after intracellular staining for Gag (Fig. 4D). The percentages of infected Jurkat cells dropped drastically from day 3 to 7, whereas no transmission was observed when AZT was added at the same time as T cells.

In conclusion, the amount, the quality of the viral particles secreted and the capacity to transmit infection to T cells all decreased between 3 and 7 dpi. On the other hand, the amount of intracellular Gag as well as the density of the viral particles per VCC increased suggesting a progressive sequestering of the virus within the cell.

### Virus-Containing Compartments can Access the Extracellular Media

**Figure 5:**
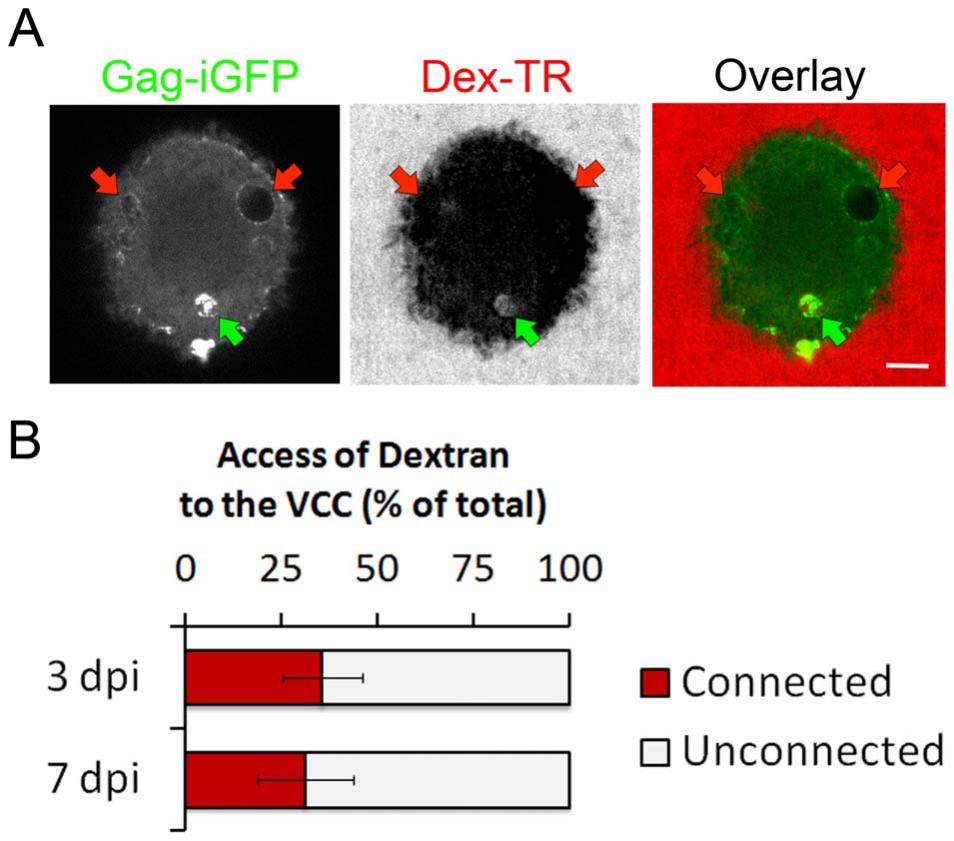
Virus-Containing Compartments connection to the plasma membrane. (*A*) Only a subset of the VCCs is rapidly accessible to Dex-TR. Confocal micrographs of living macrophages infected with HIV Gag-iGFPΔEnv virus for 3 days were imaged immediately after addition of 3 kDa Dex-TR (40 µg/ml). (*B*) Quantification of the VCCs accessible to Dex-TR at 3 or 7 dpi. Percentages of connected/unconnected VCCs according to dextran labeling were quantified at 3 and 7 dpi. About one-third of the VCCs were connected to the plasma membrane, independently of the time post infection. Histograms are means of experiments performed on 3 donors (more than 200 VCCs were analyzed for each time point).

It has been proposed that VCCs originate from the plasma membrane and could be connected to it through narrow microchannels [11], [14]. To investigate the presence and the evolution with time post infection of a VCC connection to the extracellular media on live cells, we took advantage of our capacity to image VCCs in live cells i.e. without fixation, which can be a source of artifact. Live macrophages infected with HIV Gag-iGFPΔEnv were exposed to a fluorescent fluid phase marker; a 3 kDa Dextran-Tetramethyl-Rhodamin (Dex-TR) and cells were imaged immediately after. We observed that the Dex-TR was able to access only few VCCs (Fig. 5A). Quantification of large numbers of VCCs at 3 and 7 dpi (Fig. 5B) revealed that the proportion of plasma membrane connected versus unconnected VCCs remained roughly constant with time, around one third versus two thirds, respectively.

We conclude that plasma membrane connections to VCCs allow passive diffusion of small compounds. Moreover, while the viral density within the VCCs increases with time post-infection, the proportion of VVCs connected to the extracellular medium remains constant.

### Connection of the Virus-Containing Compartment to the Plasma Membrane can be Transient

**Figure 6:**
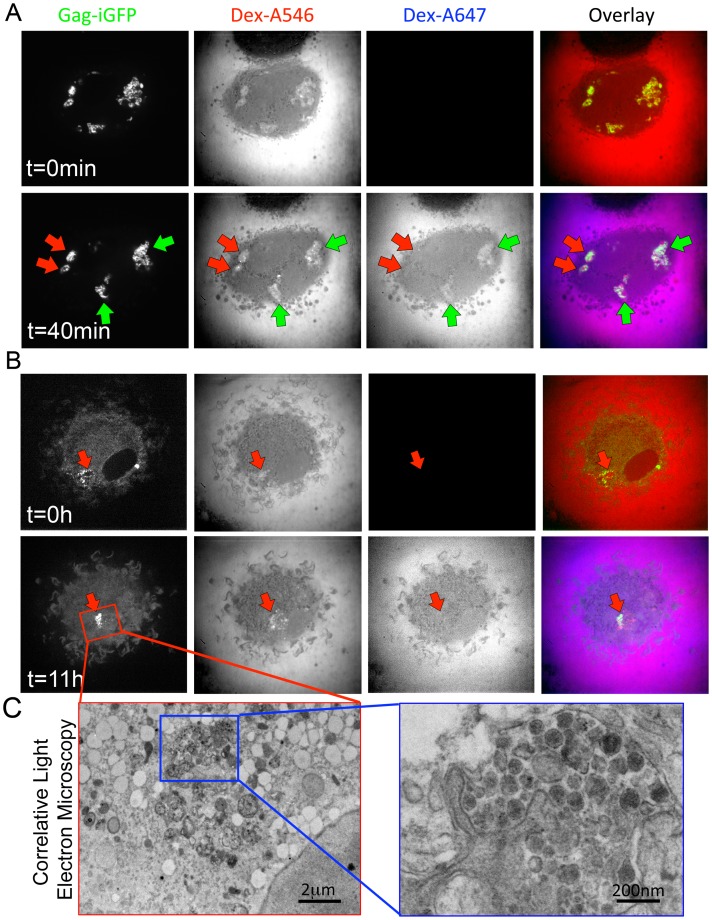
Transient connection of the Virus-Containing Compartment with the plasma membrane. (A–C) Macrophages were infected for 4 days and imaged as in Fig. 3C. (A) Imaging was first performed after addition of a 10 kD Dex-A546 (t = 0 min, upper panel). A 10 kD Dex-A647 was added 40 min later, and imaging of the same cell was carried out (t = 40 min, lower panel). Several Gag-iGFP compartments are visible and appear accessible to both dextrans (green arrows) but others remain inaccessible to second dextran (red arrows). (*B*) Confocal imaging from 4 to 5 dpi, was performed immediately after addition of Dex-A546 (t = 0 min, upper panel). A Gag-iGFP+ sparse compartment containing Dex-A546 can be seen. After 11 hours (t = 11 h, lower panel), the same cell was exposed to Dex-A647 and imaged immediately after. The Gag-iGFP+ compartment appeared denser but remained negative for Dex-A647. (*C*) Correlative light and EM of the very same cell show that the GFP signal observed in light microscopy corresponds to a *bona fide* dense VCC containing viral particles.

Our data suggest that at a given time some VCCs are connected to the plasma membrane while some are not. This raised the question of whether such connections are transiently or permanently established. To address this question, live HIV Gag-iGFP-infected macrophages were sequentially exposed to 10 kDa dextran-Alexa546 (Dex-A546) and 40 min later to 10 kDa dextran-Alexa647 (Dex-A647). Imaging immediately after each treatment (Fig. 6A) revealed that within the same cells, few Gag-iGFP+ compartments accessible to the first dextran became inaccessible to the second one (red arrows), while some other compartments remained accessible to both dextrans (green arrows). These data suggest that the connection of VCCs to the extracellular medium can be transient.

This result was further confirmed and extended by following the fate of individual VCCs for 12 h, combining time-lapse imaging of HIV-infected macrophages in our double dextran assay together with correlative electron microscopy. Infected macrophages were first exposed to Dex-A546 (t = 0 h) and then after 11 h to Dex-A647. Imaging performed immediately after dextran exposures revealed the presence at t = 0 of compartments containing low amounts of Gag-iGFP, which were positive for Dex-A546 (Fig. 6B). After 11 h, one given VCC appeared denser, was clearly accessible to the first dextran but not to the second one (Fig. 6B, see red arrow), suggesting that this compartment had lost its plasma membrane connection. Moreover, correlative EM on the very same cell established that this dense compartment contained both mature and immature viral particles (Fig. 6C).

We conclude that, with time post-infection of macrophages, access of the extracellular medium to VCC can be lost.

## Discussion

In human primary macrophages, the assembly and storage of newly synthesized HIV particles takes place in internal compartments, the VCCs [8], [9]. However, very little is known about the specific characteristics of the VCCs in primary human macrophages. The present study brings new insights regarding the dynamics of VCCs, their content, their fate and their connection with the plasma membrane.

First, by use of a replication-competent internally GFP-tagged HIV-1 virus, we approached the dynamics of the viral cycle in primary macrophages. In a previous study, macrophages infected with a tetracystein-tagged Gag HIV virus were monitored at 48 h post infection for short periods of time (up to 81 min) and found upon FlAsH labeling that Gag accumulated in internal compartments during this time window [19]. Our approach allows time-lapse microscopy of Gag in macrophages for much longer periods. We observed that one to three days after infection with a one-cycle ΔEnv virus were required to start to visualize the presence of Gag in the cytosol. The delay in Gag expression probably reflects the slow speed at which the first phases of the viral cycle takes place in primary macrophages [16]. The 14 hours delay observed between the appearance of cytosolic Gag and the presence of Gag+ compartments is most likely due to the time needed for Gag to reach and/or form the compartments, which we showed by ultrastructural analysis were indeed VCCs. With both Env- and Env+ viruses, cytosolic phase precedes Gag+ compartment detection, suggesting that the formation of VCCs was Env-independent. Moreover, electron microscopy of macrophages infected with HIV-1 ΔEnv also shows similar virus-containing compartments (not shown). Finally, the Gag+ VCCs were rather large, usually in the µm range (0.5 to 5 µm) as previously estimated at the ultrastructural level [14].

From day 3 post-infection, macrophages exhibited two main categories of VCCs with respect to their Gag-iGFP content: the “sparse” ones had low amounts, while the “dense” ones appeared totally filled. In the sparse VCCs, we observed movements of Gag+ structures with high velocity suggesting that once freed in the lumen of the VCC, the virions were relatively free to move. In contrast, in dense VCCs virions appeared rather immobile probably in part due to the their high density but additional factors may contribute to their stillness. Budding profiles were clearly observed at the limiting membrane of both sparse and dense VCCs. Therefore, VCCs represent, in all likelihood, the site of viral assembly in primary macrophages. Finally, our data suggest that VCCs accumulate newly formed virions in their lumen and progressively fill up with them.

Second, we show using immunobloting, confocal and electron microscopy that VCCs accumulate in macrophages, become denser, and that the amount of p24 Gag present in infected macrophages increases over time post-infection. In parallel, the amounts of secreted viral particles diminish with time and their infectivity as well. Furthermore, the capacity of infected macrophages to transmit the virus to T cells added in co-culture also drastically decreases over time. Taken together these data suggest that over time post-infection, viral particles tend to stay longer within VCCs where they may slowly lose their infectious properties. The idea that retention and packing may damage the viral particles is supported by the known fragility of HIV-1 [20], [21]. The retention of the virions within the VCCs might be mediated at least in part by the HIV-1-induced expression of tetherin in macrophages, a protein found enriched at the level of the VCCs ([22] and Fig. S5), which has been proposed to tether virions to the VCC limiting membrane in macrophages [22]. Moreover, tetherin could also contribute to the relative stillness of the viral particles observed in dense VCCs.

Third, we show that one third of the VCCs are connected to the plasma membrane and that such connections can be lost. These experiments are the first to our knowledge that addressed the plasma membrane connection in live cells instead of electron microscopy of fixed samples, which may generate artifacts. The calculation of one third of the compartments being accessible to dextran is in line with our previous estimation that roughly 20% of the VCCs identified by EM were accessible to ruthenium red [10]. In another study, similar experiments showed that 20 to 80% of the VCCs were accessible to the dye, depending on the donor [11]. Such plasma membrane connections could favor rapid exchange of protons and explain the previously reported neutral pH of VCCs [10]. In contrast to the conclusions from our experiments performed by live cell imaging, it has been recently reported that small dextran did not access VCCs [23]. However, the use of non-fixable dextran in these experiments where cells were fixed, permeabilized and stained for Gag probably explains the incapacity to visualize fluorescent dextran in VCCs. The same study reported the failure to reveal plasma membrane connection by electron microscopy using cationized ferritin instead of ruthenium red, but the large size of this molecule (roughly 450 kDa) and its capacity to form large aggregate probably explain its failure to access VCCs, thus indirectly confirming previous studies regarding the limited width of the VCC connections [14], [24].

Our study also reveals that at the steady state, VCCs appear largely heterogeneous in size and contents. Previous EM studies clearly illustrated this heterogeneity in terms of density of virions, ranging from very low to very high [5], [6], [10], [11], [14], [25]. Our demonstration that VCC connections to the plasma membrane are transient tends to reconcile previously reported discrepancies about the existence of a VCC connection to the extracellular milieu. Taken together our data are compatible with ultrastructural studies suggesting that VCCs possess a complex architecture [14], [24], [26], [27] made of intricate and complex tubular membranes connected to the external milieu through narrow tube-like structures [24] or conduits [26].

It has been proposed that VCCs could represent endosomes containing internalized viral particles produced by neighboring cells [28] rather than internal compartments filled with newly formed viral particles. However, several lines of evidence preferentially support the later idea: 1) We and others have obtained numerous EM profiles showing viral buds at the limiting membrane of the VCCs and immature viral particles inside their lumen [5], [6], [10]. 2) We previously reported that macrophage exposure to HIV-1 at high MOI led to the internalization of virions into the endosomal pathway and their rapid degradation in agreement with the seminal work of Marechal et al. [29]. Importantly, lysosomal markers such as Lamp1 are consistently absent from macrophage VCCs in our hands. 3) Live cell recording of macrophages for several days, starting immediately after exposure to HIV Gag-iGFP reveals that fluorescent viral particles are internalized and rapidly disappear after a few hours (not shown), suggesting that the wide majority of the viral particles added are initially internalized and degraded. Newly synthesized Gag-iGFP requires at least one day to be visualized in the cytosol as a diffuse staining and consistently occurs before the VCCs can be seen (see Video S1).

**Figure 7:**
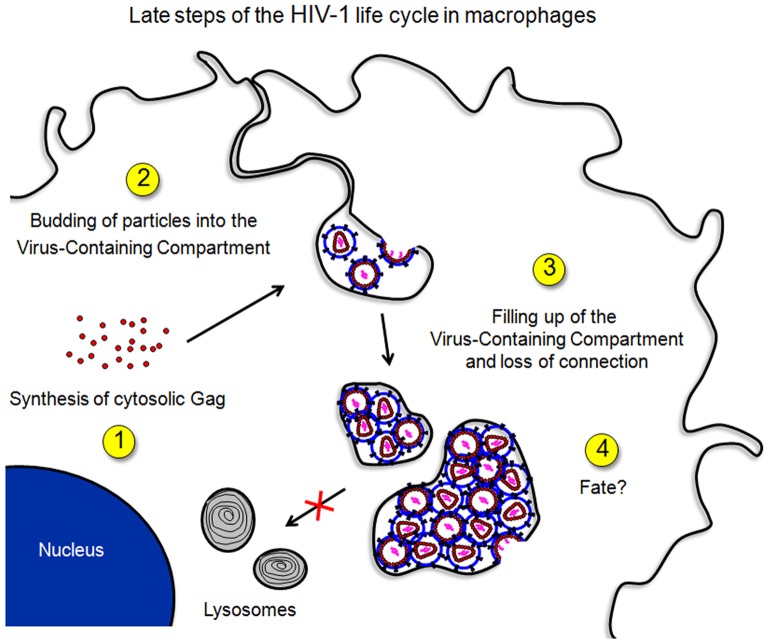
Schematic representation of HIV-1 production in primary macrophages. (1) Following entry of HIV, it takes 1 to 3 days before cytosolic Gag becomes visible as a diffuse pattern. (2) VCCs are visible usually 0.5 to 1 day later. Transient connections to the plasma membrane are present via narrow microchannels. (3) Viral particles accumulate in VCCs filling them with time. (4) VCCs may reside inside the cell or travel toward the plasma membrane where they can release their viral content in the extracellular space.

Integrating the present work with the literature allows us to construct a hypothetical model for the dynamics of VCCs (Fig. 7). Upon HIV-1 infection of macrophages, Gag is synthesized and accumulates in the cytosol before promoting the formation of Gag+ internal compartments probably originating from the plasma membrane. Viral assembly and budding occur at the limiting membrane of this compartment. Newly formed particles fill up the luminal space of the VCCs overtime, and narrow connections between VCCs and the plasma membrane are lost. While VCCs do not seem to fuse with lysosomes, part of them may reside in the cytosol for long periods of time where they may accumulate virions that will lose their infectious capacity with time. Finally, the VCCs appear to be driven toward the plasma membrane where they release their virions into the extracellular space or to neighboring cells, through a mechanism involving the KIF3A complex [13].

The regulation of the viral release remains so far completely obscure. Since HIV-1-infected macrophages are long-lived, the few VCCs - and thus virions - they can release may contribute to viral persistence *in vivo*. Future work in primary macrophages will aim at detailing, at the molecular level, how the release of viral particles is regulated.

## Methods

### Cells

Buffy coats were obtained from adult healthy donors (Saint-Antoine Crozatier Blood Bank, Paris, France) where all donors signed informed consent allowing the use of their blood for research purpose. Monocytes were isolated by positive selection using CD14+ Microbeads (Miltenyi) and differentiated into macrophages for 7 days in complete medium supplemented with M-CSF (25 ng/ml final, ImmunoTools). Reporter cell lines were obtained through the NIH AIDS Research and Reference Reagent Program; GHOST from VN Kewal Ramani and DR Littman; and TZM-bl from JC Kappes, X Wu and Tranzyme Inc.

### Antibodies, Plasmids and Reagents

Goat polyclonal antibodies against p24, mouse monoclonal against human CD81 (clone TS81, Abcam), CD9 (Santa Cruz), GFP living color (Clontech), Actin (MP Biomedicals), Lamp1 (Pharmingen) and secondary antibodies conjugated with Alexa Fluor 488 or 647 (Invitrogen), Cy3 or Cy5 (Jackson) were used. Anti-Env (2G12) and anti-p17 (4811) were obtained through the NIH AIDS Research and Reference Reagent Program. The mAb specific for the Matrix (p17) was obtained from the National Institute for Biological Standards and Control (NIBSC) centralized facility for AIDS reagents (ARP342, from RB Ferns and RS Tedder). Dextran 3 kDa conjugated to Tetramethyl-Rhodamin, Dextran 10 kDa conjugated to Alexa Fluor 546 or 647 (Molecular Probes) were used at 40 µg/ml.

HIV-1 p24 was measured using the Innotest HIV Ag mAb Screening kit (Ingen). The provirus pUC NL4-3 Gag-iGFP was a kind gift from B. Chen [15]. To generate an R5-tropic virus, its Gag-iGFP coding sequence was cloned into the pBR–NL4-3 vector backbone. Then, the V3-loop V92th014.12 was inserted instead of the NL4-3 one [27]. The resulting virus is referred to as HIV Gag-iGFP. We also derived an *env*-defective version of the provirus, which does not express Env nor Vpr and is referred to as HIV Gag-iGFPΔEnv. The plasmid p-VSV-G (Becton Dickinson) was used for pseudotyping.

### Virus Preparations and Infections

NL4-3 AD8 (NLAD8) [30], HIV Gag-iGFP and HIV Gag-iGFPΔEnv were produced by transfection of the corresponding proviral cDNA in HEK 293T cells (ATCC number: CRL-11268) by PEI precipitation. Pseudotyping was eventually achieved by co-transfection of pVSV-G. Supernatant was harvested 72 hours after transfection, and ultracentrifuged at 100,000g for 90 min at 4°C. Pellets were resuspended in 2% BSA in PBS. Virus preparations were titrated by infecting the GHOST reporter cell line and infectivity was measured 24 hours post-infection by flow cytometry. For infectivity assays, virion-containing supernatants were normalized for equal amounts of p24 and infectious titers were determined using TZM-bl indicator cells as described [31]. For macrophage to T cell transmission assays, macrophages (250.000 cells/well) were infected with VSV-g-pseudotyped NL4-3 at MOI 1.5. Three or seven days after infection, macrophages were extensively washed with PBS and 75.000 Jurkat T cells were then added to each well. AZT was added at 0 hour (as control) or 24 hours after the beginning of the co-cultures. At 48 h, Jurkat T cells were collected, washed with PBS and fixed with PFA 1%. Cells were permeabilized, stained for Gag with monoclonal antibody KC57-FITC (Beckman-Coulter), and analyzed by flow-cytometry in a BD Accuri C6 flow cytometer. Percentage of infected cells refers to the percentage of Gag+ events inside a tight gate on Jurkat population defined on a FSC vs SSC plot. By surface CD3 staining, we established that events in such gate are over 97% CD3+.

### Immunoblotting

Cells were lysed in 0.5% NP40 lysis buffer +2 mM PMSF for 30 min at 0°C. Cell lysates were loaded on 4–12% Bis-Tris Acrylamid gels (NuPAGE system, Invitrogen), transferred to a PVDC membrane, blocked and immunoblotted in PBS 0.1% Tween20 and 5% milk.

### Immunofluorescence, Electron Microscopy and Live Imaging

Cells were fixed in 4% paraformaldehyde, permeabilized and stained in 0.2% BSA, 0.5% Saponin in PBS. Samples were mounted on coverslips in Dapi Fluoromount G (SouthernBiotech) and imaged on a Nikon Ti Inverted Microscope fitted with a confocal A1R system.

HIV-1-infected macrophage samples were prepared for immuno-electron microscopy as previously described [10]. For epon embedding, HIV-infected macrophages cultured on glass coverslips were fixed with 1.6% glutaraldehyde in 0.1 M sodium cacodylate buffer (pH 7.4) for 1 h at RT. After washing in 0.1 M sodium cacodylate buffer, the cells were postfixed with 1% osmium tetroxide in same buffer for 1 hour, dehydrated through a series of ascending concentrated ethanol, embedded in epoxy resin and then processed for ultrathin sectioning. Sections were observed and photographed under a Philips CM120 electron microscope at 80 kV (FEI, Eindoven, The Netherlands). Digital acquisitions were made with a numeric camera Keen View and analyzed using the Item software (Soft Imaging System, Munster, Germany). Long time-lapse movies (days) were acquired using a Nikon Biostation IM-Q. Fast imaging was performed on a Nikon Ti Inverted Microscope fitted with a video-rate confocal system consisting of a spinning disk confocal head (Yokogawa). Images were collected using a 100× oil immersion objective with a numerical aperture of 1.4 with a HQ2 camera (Photometrics). FRAP experiments were performed on this microscope with a second 491 nm excitation laser using maximal power voltage.

### Statistical Analysis

The statistical significance of the differences was determined from the means and standard deviations by using the Student’s t-test.

## Supporting Information

Figure S1 **Characterization of HIV Gag-iGFP in macrophages.** (A) Primary macrophages were infected with HIV WT or HIV Gag-iGFP both being pseudotyped with VSV-G at a MOI of 2. Amounts of p24 released in the culture supernatant were determined by ELISA at various times after infection. The graph is representative of results obtained with two donors and values are means +/− SD of quadruplicates. (B) Confocal micrographs of macrophages infected with HIV WT or HIV Gag-iGFP for 7 days were prepared for immunofluorescence and stained for the indicated markers. Bar 10 µm. (C) Confocal micrographs of macrophages infected with HIV NLAD8 or HIV Gag-iGFP were fixed 3 dpi and stained for the indicated markers. Bar 5 µm. (D) Infectivity was assayed using normalized amounts of virions (2 ng of p24) produced by macrophages infected for 7 days on the TZM-bl reporter cell line. Values are means +/− SD of quadruplicates. (E) Electron microscopy of macrophages infected with HIV Gag-iGFP for 7 days. (F) Immunoblot analysis of the cell lysates of macrophage infected for 7 days revealed with anti-p24 (KC57-FITC), anti-GFP or anti-Actin antibodies.
(TIF)

Figure S2 **Free GFP is not generated in the cytosol of HIV Gag-iGFP-infected macrophages.** Confocal micrographs of macrophages infected with a lentivector encoding for GFP only or with HIV Gag-iGFP. Cells were fixed 7 dpi and stained with Dapi.
(TIF)

Figure S3 **Dynamic imaging of the HIV cycle in macrophages.** Macrophages infected with HIV Gag-iGFPΔEnv virus were imaged from 1 to 8 dpi. To minimize photo-cytotoxicity, images were acquired every 15 min with an epifluorescent Biostation microscope. Here are presented 3 snapshots from the movie at the indicated times post infection. Epifluorescent and corresponding transmission images are presented.
(TIF)

Figure S4 **Gag accumulates into macrophages overtime.** Macrophages from 4 donors were infected with NL4-3 ΔEnv at MOI 1 and washed 8 h after. At day 2 and day 6 post-infection, medium was replaced to monitor secretion of p24 from day 2 to 3 and 6 to 7. Cell viability was evaluated by CellTiter-Glo and intracellular p24 was measured in matched lysates. p24 data were normalized for cell viability. The relative proportion of secreted (white bars) and intracellular p24 (grey bars) was evaluated at both time points.
(TIF)

Figure S5 **Tetherin is present in Gag+Env+ compartments of infected macrophages.** Confocal micrographs of macrophages infected for 6 days with NLAD8 were fixed and stained for Gag (KC57), Env (2G12) and Tetherin (NIH 11721). Bar: 10 µm.
(TIF)

Video S1 **Time-lapse fluorescent microscopy of infected-macrophages.** Macrophages were infected at time 0 with HIV Gag-iGFP. Images were acquired every 15 min from 1 to 8 days on a Nikon biostation. The movies presented are from 2 to 4 dpi. The right panel presents the GFP epifluorescent channel while on the left panel, transmission images have been super-imposed to the GFP fluorescence.
(AVI)

Video S2 **Time-lapse of the various kinds of VCC.** Macrophages were infected with HIV Gag-iGFP ΔEnv for 5 days. Time-lapse spinning disc confocal microscopy of the GFP fluorescence was recorded at 1 image/50 msec at 37°C in 5% CO_2_ for 10 sec. Half-empty (“sparse”) compartments exhibit internal movements of Gag+ structures of high velocity.
(AVI)

Videos S3 **Fluorescent recovery after photobleaching.** Macrophages infected for 4 days with HIV Gag-iGFP ΔEnv were imaged before photobleaching for 5 sec, bleached (20 ms). Video S3 presents a recording upon photobleaching of the part of the VCC (within the red circle of Fig. 2A).
(AVI)

Videos S4 **Fluorescent recovery after photobleaching.** In Video S4, photobleaching was performed in the cytosol (within the red circle of Fig. 2B). Rapid fluorescence recovery is observed in the later case but not in the former. The arrow points to the area that is going to be bleached and the movie stops for a few seconds just before bleaching.
(AVI)

Video S5 **Time-lapse video of a 3D reconstruction of a homotypic fusion of two VCCs.** Macrophages were infected with HIV Gag-iGFP ΔEnv for 4 days. Time-lapse spinning disc confocal microscopy of the GFP fluorescence was recorded at 1 image/5 min at 37°C in 5% CO_2_.
(AVI)
