# Supplementary figures and images for: Dynamics of HIV-Containing Compartments in Macrophages Reveal Sequestration of Virions and Transient Surface Connections
Source: PLoS One. 2013 Jul 29;8(7):e69450. doi: 10.1371/journal.pone.0069450 (PMC3726685; doi:10.1371/journal.pone.0069450)

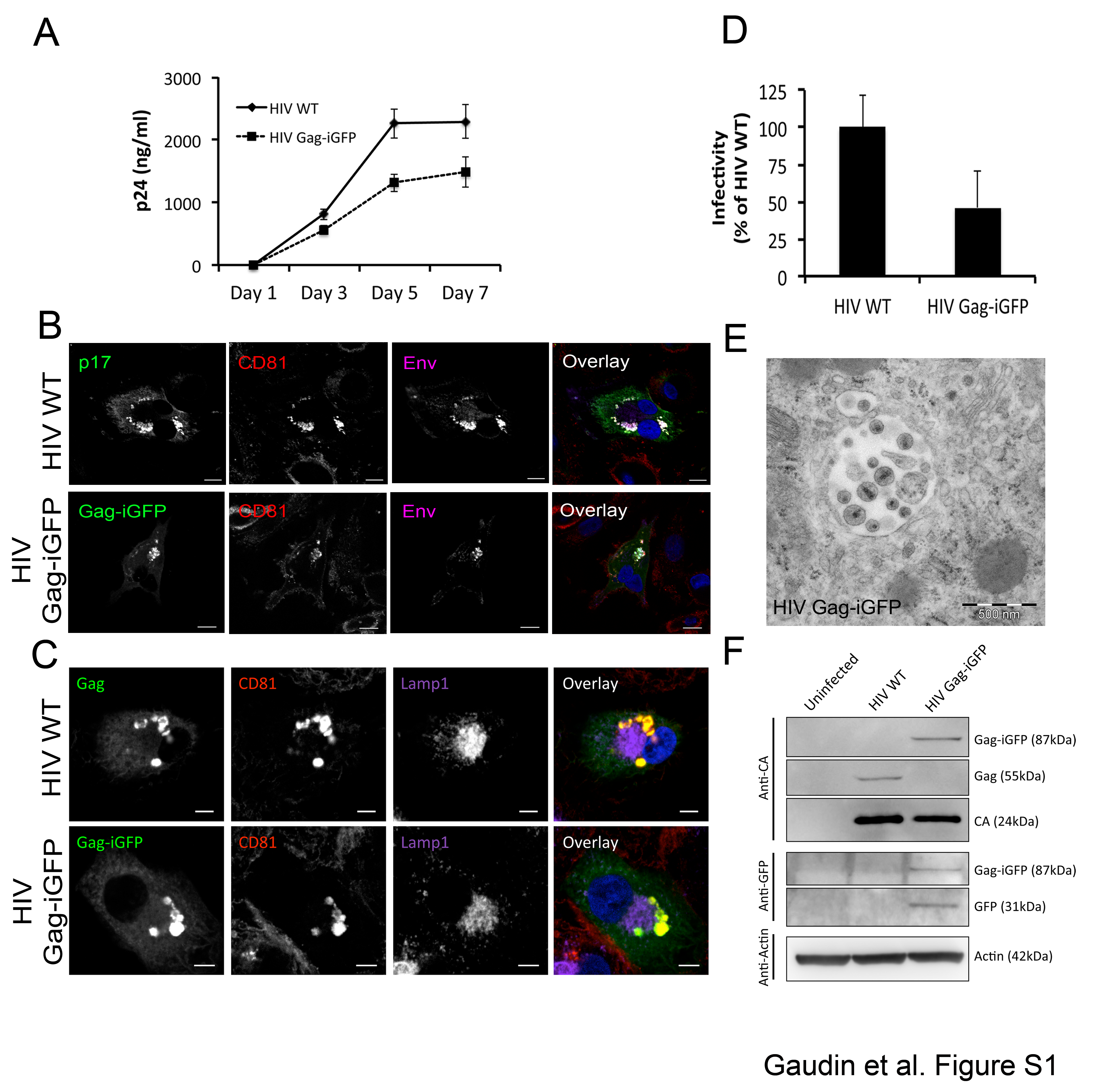

Supplement: Figure S1 — Characterization of HIV Gag-iGFP in macrophages. (A) Primary macrophages were infected with HIV WT or HIV Gag-iGFP both being pseudotyped with VSV-G at a MOI of 2. Amounts of p24 released in the culture supernatant were determined by ELISA at various times after infection. The graph is representative of results obtained with two donors and values are means +/− SD of quadruplicates. (B) Confocal micrographs of macrophages infected with HIV WT or HIV Gag-iGFP for 7 days were prepared for immunofluorescence and stained for the indicated markers. Bar 10 µm. (C) Confocal micrographs of macrophages infected with HIV NLAD8 or HIV Gag-iGFP were fixed 3 dpi and stained for the indicated markers. Bar 5 µm. (D) Infectivity was assayed using normalized amounts of virions (2 ng of p24) produced by macrophages infected for 7 days on the TZM-bl reporter cell line. Values are means +/− SD of quadruplicates. (E) Electron microscopy of macrophages infected with HIV Gag-iGFP for 7 days. (F) Immunoblot analysis of the cell lysates of macrophage infected for 7 days revealed with anti-p24 (KC57-FITC), anti-GFP or anti-Actin antibodies. (TIF) [file pone.0069450.s001.tif]

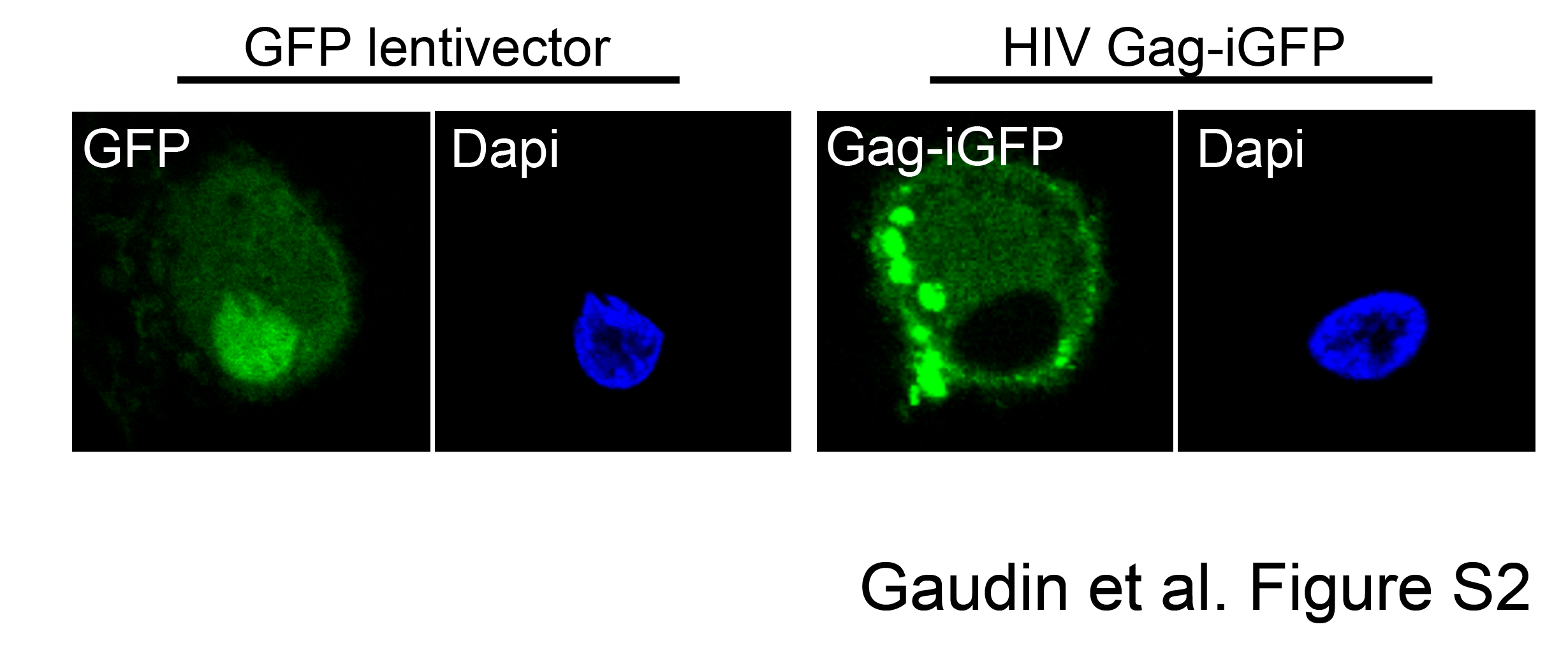

Supplement: Figure S2 — Free GFP is not generated in the cytosol of HIV Gag-iGFP-infected macrophages. Confocal micrographs of macrophages infected with a lentivector encoding for GFP only or with HIV Gag-iGFP. Cells were fixed 7 dpi and stained with Dapi. (TIF) [file pone.0069450.s002.tif]

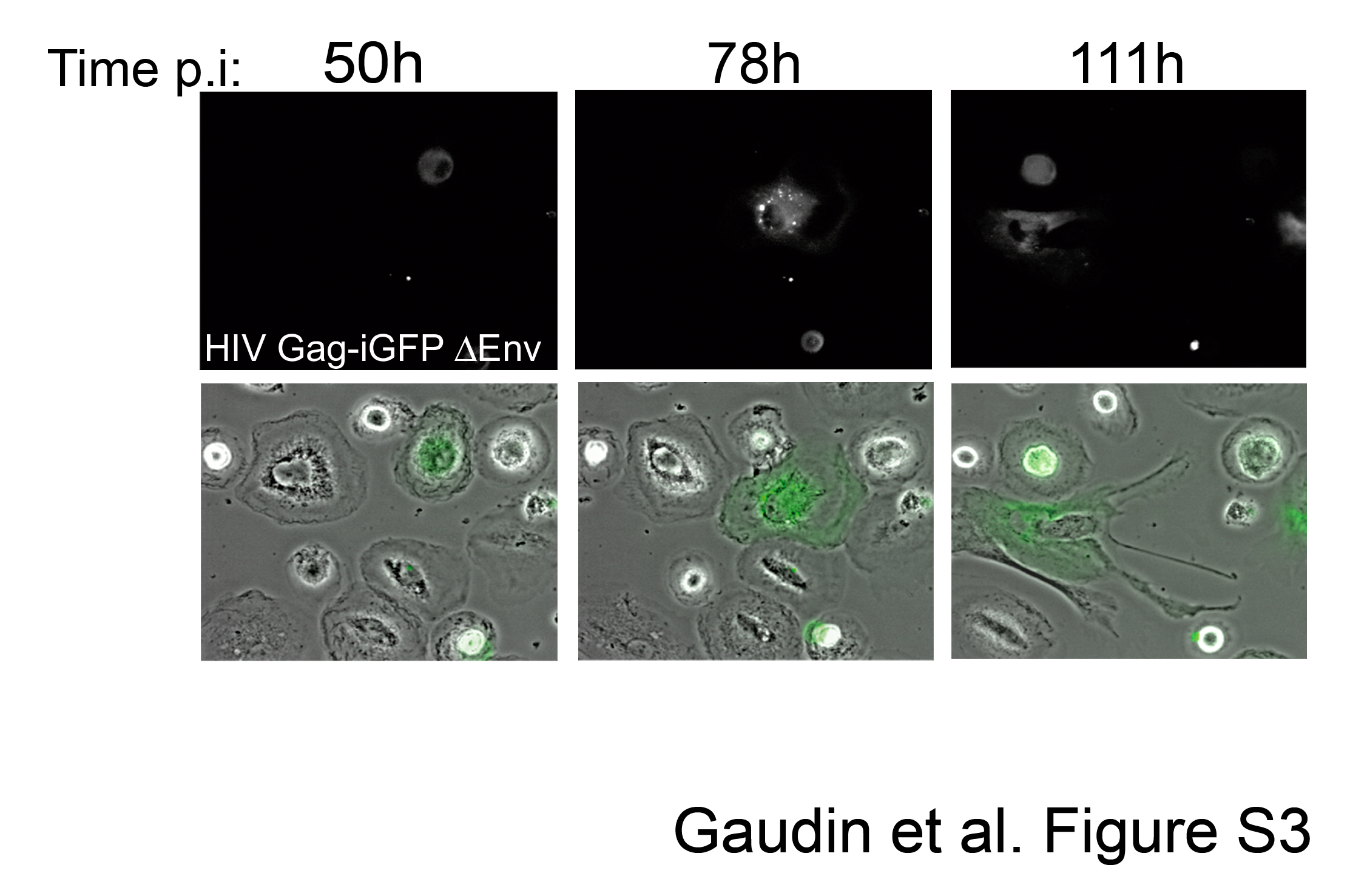

Supplement: Figure S3 — Dynamic imaging of the HIV cycle in macrophages. Macrophages infected with HIV Gag-iGFPΔEnv virus were imaged from 1 to 8 dpi. To minimize photo-cytotoxicity, images were acquired every 15 min with an epifluorescent Biostation microscope. Here are presented 3 snapshots from the movie at the indicated times post infection. Epifluorescent and corresponding transmission images are presented. (TIF) [file pone.0069450.s003.tif]

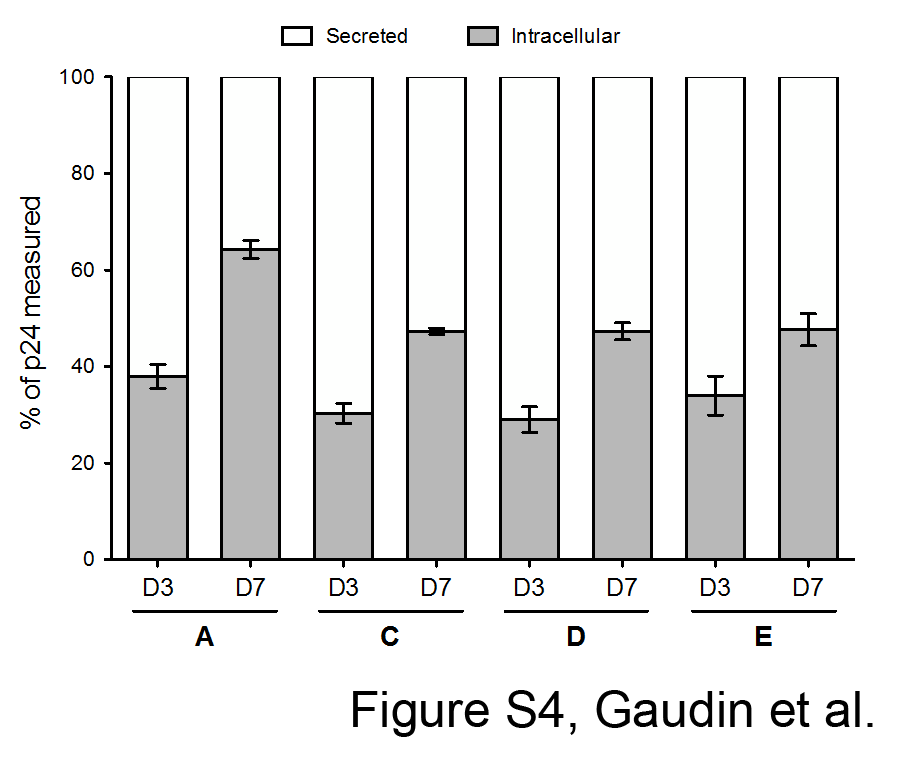

Supplement: Figure S4 — Gag accumulates into macrophages overtime. Macrophages from 4 donors were infected with NL4-3 ΔEnv at MOI 1 and washed 8 h after. At day 2 and day 6 post-infection, medium was replaced to monitor secretion of p24 from day 2 to 3 and 6 to 7. Cell viability was evaluated by CellTiter-Glo and intracellular p24 was measured in matched lysates. p24 data were normalized for cell viability. The relative proportion of secreted (white bars) and intracellular p24 (grey bars) was evaluated at both time points. (TIF) [file pone.0069450.s004.tif]

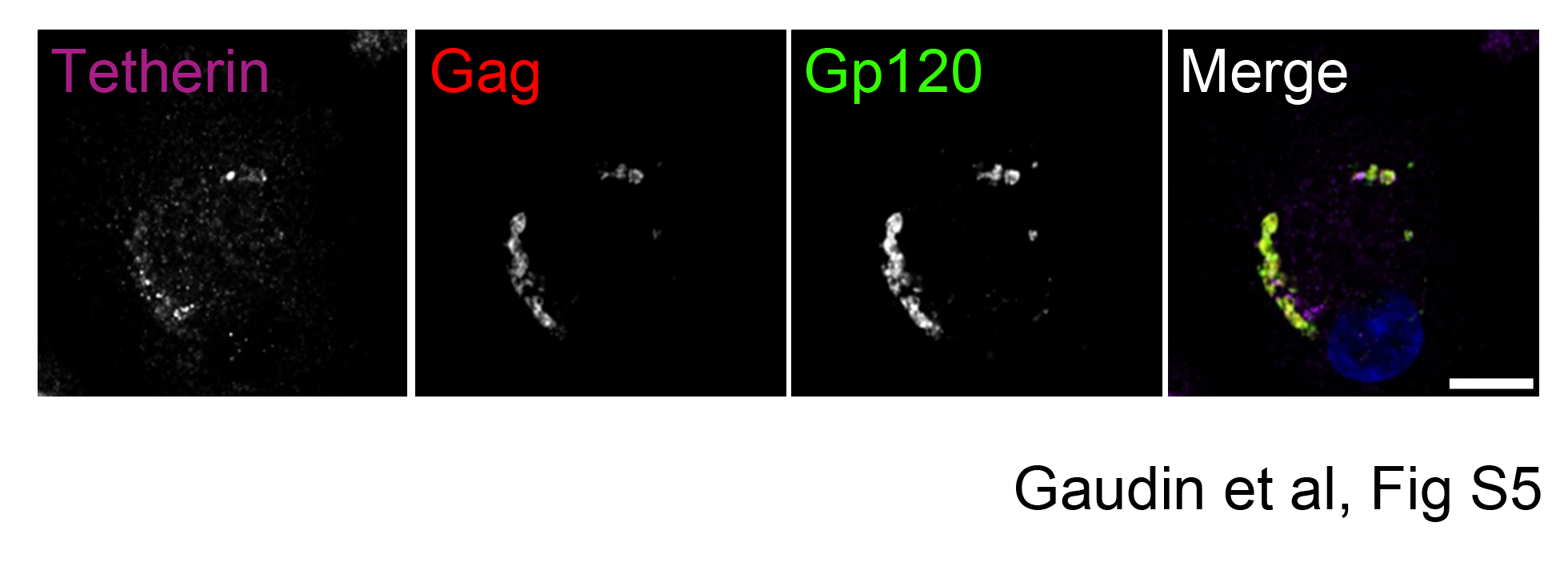

Supplement: Figure S5 — Tetherin is present in Gag+Env+ compartments of infected macrophages. Confocal micrographs of macrophages infected for 6 days with NLAD8 were fixed and stained for Gag (KC57), Env (2G12) and Tetherin (NIH 11721). Bar: 10 µm. (TIF) [file pone.0069450.s005.tif]
